# Beverage intake among Jamaican School Children: An analysis across child and school environment levels

**DOI:** 10.1371/journal.pone.0354352

**Published:** 2026-08-03

**Authors:** Emile Charles, Suzanne Soares-Wynter, Shu Wen Ng

**Affiliations:** 1 Department of Nutrition, Gillings School of Global Public Health, University of North Carolina - Chapel Hill, Chapel Hill, North Carolina, United States of America; 2 Caribbean Institute for Health Research, The University of the West Indies - Mona, Kingston, Jamaica; 3 Carolina Population Center, University of North Carolina - Chapel Hill, Chapel Hill, North Carolina, United States of America; Oswaldo Cruz Foundation: Fundacao Oswaldo Cruz, BRAZIL

## Abstract

Sugar-sweetened beverages, a subset of ultra-processed foods, are consumed frequently by children in Jamaica. Given the abundance of time children spend in school and the evidence for school-related determinants of dietary intake, school food environments can be key influencers of children’s beverage consumption and drive their dietary behaviors and preferences. 907 primary school students (ages 7–11) in Jamaica were surveyed on their demographic information, nutrition-related preferences, knowledge, and consumptions patterns of sweetened and unsweetened beverages in 2018. School environment audits, documenting internal and external factors from the 30 schools which children attended, were completed at the same time. A two-part model was applied that considered children’s probability of consuming each beverage type and the amount they consumed. Models accounted for both child-level characteristics and school food environment measures. Descriptive statistics revealed a large proportion of non-consumers of water (20.9%) and unsweetened beverages (19.8%). Collectively, child-level factors contributed to the modeling of all beverage types and external school environment measures were highly significant to the sweetened beverage model fit. External school measures were jointly associated with sweetened beverage consumption volume while unsweetened beverage intake is more likely impacted by classroom rules, school wide policies, and the internal school environment. Results support the inclusion of beverage regulations, both the promotion of unsweetened and the de-incentivization of sweetened drinks, in Jamaica’s recently approved national school nutrition policy.

## Introduction

While the prevalence of overweight and obesity have risen globally, these challenges are particularly acute in the Caribbean.[1–3] Low levels of physical activity, a lack of health-promoting nutrition policies, and a food environment oversaturated with nutrient-poor products all contribute to an elevated prevalence of obesity among Caribbean populations.[1,4] Commercial determinants, such as the excessive import of ultra-processed foods and sugar-sweetened beverages, have also contributed to a regional rise in nutritional non-communicable disease, including cardiovascular disease and diabetes.[1,5,6] Furthermore, poverty and inequalities in education have been shown to drive higher rates of obesity across the region.[7,8]

In Jamaica, specifically, investigations have been conducted to describe population level obesity prevalence and its determinants. From 2000 to 2020, the country experienced more than a 10% increase in adult obesity prevalence.[6,9] Among children and adolescents, rates of both overweight and obesity have more than doubled in the same time period.[9] The most recent data suggests a 13.9% prevalence and 25.3% among children and adults, respectively.[10]

Researchers have tried to understand key influencers of childhood obesity that are embedded into the spaces in which children live, learn and play. At the child and household level, there is evidence that parental income, child food and beverage knowledge, and parental guidance all influence children’s dietary habits. At the school-level, where children spend much of their time, the large presence of ultra processed food and drinks, limited outdoor recreational space, and influence of external vendors have been shown to play a significant role.[11]

As a subset of ultra processed foods, sugar-sweetened beverages (SSB) are a major contributor to obesity and cardiometabolic conditions, and the consumption of such products by adolescents are higher in Latin American and Caribbean region than any other area of the world.[12–16] Since 1990, Jamaican youth have consistently consumed, on average, more than 10 SSB servings per week, an intake greater than the regional mean.[15,17] Previous work has shown that children and adolescents who consume SSB are more likely to be obese and consume other types of ultra-processed products.[18,19]

Data have shown that SSB excise taxes, food labeling policies, and other policies to decrease the presence of SSB in food environments can reduce children’s consumption, and consequently reduce excess weight.[20–23]

In June 2025, Jamaica’s Parliament approved the new national school nutrition policy, jointly developed by the Ministry of Education, Skills, Youth and Information and the Ministry of Health and Wellness.[24]. One focus of this policy will be regulating nutritional standards within school and managing the internal and external food environments.[25] Defined as all of the spaces, infrastructure, and conditions inside and around school premises where food is available, obtained, purchased, and/or consumed, school food environments (SFE) are instrumental in determining what and how children consume when at school.[26]

In this secondary analysis, we will be assessing the associations between our primary exposures, child demographics and school environment factors, and our primary outcomes, sweetened beverage, unsweetened beverage, and water intake. This investigation will allow for an analysis across multiple levels of determinants, as well as identification of where school nutrition interventions may be most effective. This secondary analysis will further assess which components of Jamaican SFE are most influential on primary children’s beverage consumption patterns, helping to inform and enrich school nutrition policies and guidelines.

## Methods

### Data

This was a cross-sectional study of children grade 3–5 attending 30 different primary schools in the capital city Kingston and neighboring Saint Andrew parish in Jamaica conducted in 2018. Schools were selected from a list of 747 registered schools across the two parishes and using a probability proportional to number of students. Primary schools were focused on given the critical nature of early childhood in forming dietary habits.[27,28] This area provided a sample of schools with varying sizes and socio-economic traits. Both public and private schools were randomly selected from a national listing using a multi-stage stratified sampling technique to first be involved in an environmental audit.

For development and training of the audit instrument prior to the study, audit points/variables were collated from senior nutritionists’ observations in 5 non-study schools. Each data collector then conducted duplicate audits and results were tested for inter- and intra-observer comparisons. Data collectors were considered trained when audit results were matched 100%.

In both training and data collection processes, two observers completed audits at each school individually, and results were compared. If there were discrepancies between the observations, study team members would conduct a repeat audit together. Results compared for inter- and intra-observer consistency, as well as completeness. Unresolved differences between surveyors were adjudicated through discussions with Jamaican research partners and schools. During the study, each data collector compared 5 school audits with senior nutritionists as the reference standard. A match of 95% inter-observer was considered acceptable.

This process was done to document all visible features of the internal built environment and existing administrative frameworks within the schools. The audit tool was created with an emphasis on the food and physical activity spaces accessible to children. Survey items were largely adapted from the ISCOLE school environment tool with additional questions added for a Jamaican context, such as questions relating to street vendors, tuck shops, and vending machines.[29]

The external school food environment was limited to the area within and along the school external perimeter, and those readily visible (approximately 100m) from school boundaries. School audit data for the study was collected around or during lunch time on school days from November 1 – December 31, 2018.

Among the 30 schools, classrooms were randomly sampled such that every child in a single from each school were included in the study. In Jamaica, children stay in their classroom for the school day, while teachers move between classrooms. This sampling strategy allowed consistent follow up with study participants. Children were given informed consent and survey forms in school to bring home for parents/guardians to complete and then return to school. Where possible, parents were also contacted via telephone to obtain any missing information.. All child measures, including dietary recall interviews, were done during the same two-month period that environmental audits were completed.

Children were asked to describe all beverages, fruits and vegetables consumed within the 24-hour period prior to the time of the interview. A structured, multi-pass approach was used to systematically recall all items including details about beverage brands, amounts, preparation method, source of item (e.g., from home or school) and time consumed. Food models and prompts were used to help with recalls. Recalls were administered by study team member in school during an approved break period.

Survey materials were pre-tested to check children’s abilities to describe items consumed compared to direct observations made by teachers or parents. Each consented child’s parent/guardian provided their child’s date of birth, grade, sex, and information about parental education and household. Given the young study population, the recalled dietary items were limited to avoid overburdening participants, including only fruits, vegetables, and beverages.

Data was accessed and analyzed from August 1, 2023 – May 1, 2025 for the purposes of this retrospective secondary analysis. All participant data was anonymized prior to analysis, and authors did not have access to information that could identify individual participants during or after data collection. Ethical approvals were obtained from the Institutional Review Board at the University of North Carolina at Chapel Hill (IRB# 18–2244), the University of the West Indies –Mona Campus Ethics Review Board (Jamaica) (IRB# ECP 198, 17/18) and the Jamaican Ministry of Education, Youth, and Information. Informed consent was obtained in written form from the students’ parents or guardians prior to each student’s participation. The study was also explained to children, and their assent was obtained prior to any measurements being conducted.

The original study occurred in Jamaica and Barbados, collecting information on school environments across the two countries, with an additional child-level component in Jamaica.[30] The objectives of the original study were as follows: to examine the associations between unhealthy dietary habits and physical activity with oversight and risk of disease; to evaluate socioeconomic, demographic, and environmental exposures; and to identify barriers and facilitators to healthy child development. The original cross-sectional study assessed children’s nutritional status through evaluation of the school environment audits and surveys with SFE stakeholders (school administrators, street vendors, parents).[30]

### Outcomes and exposures

The primary outcomes of this analysis were sweetened beverage intake, unsweetened beverage intake, and plain water intake. Sweetened beverages include bagged syrup-based drinks, flavored milks, flavored waters, food drinks, juice drinks and ice teas, sodas, diet sodas, sport drinks, and sweetened mixed drinks. Unsweetened beverages included plain water, plain milk, 100% fruit juice, and coconut water.

Covariates of the study were at the child, internal school environment, and external school environment levels. At the child level, these included gender, age, beverage knowledge/awareness, family income, and parental education level. Beverage knowledge was assessed using a child survey question asking children to indicate the healthier product when presented with water and a bagged drink. Family income was assessed using a parental survey question, indicating whether the collective family income was above or below the 2018 Jamaican national poverty line (JA $7000/week, or JA $364,000/year, or USD $2800/year).[31] Parental education level was similarly completed by parents/guardians using the parental survey, asking the highest level of education achieved from the following options: no formal education, primary school, all-age school, secondary/high school, technical/vocational school, or tertiary, college/university. At the school level, covariates included both internal and external school environment measures. The internal environment is defined as the elements within school grounds. The external environment is defined as elements off school grounds, yet readily visible (roughly within 100 meters). Measures of the internal school environment included school category, number of industry marketing elements and sponsorships/donations, number of sweetened beverage brands, number of water brands, number of school meal related equipment, spaces, and school-sponsored vending on campus, presence of indoor and outdoor drinking apparatuses, number of physical activity equipment and spaces, number of green and non-physical activity related spaces, number of child and pedestrian safety elements, number of sanitation, trash, and recycling apparatuses, and classroom policy on drinking water in the room. The external school environment measures included the presence of nearby urban residences, the presence of nearby paved walkways/sidewalks, the presence of nearby school vendors (individuals selling food whose offerings vary), the presence of nearby food stalls (semi-permanent structures from which food is sold and whose offerings are more consistent), the presence of nearby cook shops, the presence of nearby supermarkets, and the presence of nearby restaurants/fast food/sit down establishments.

### Analytic sample

**Fig 1:**
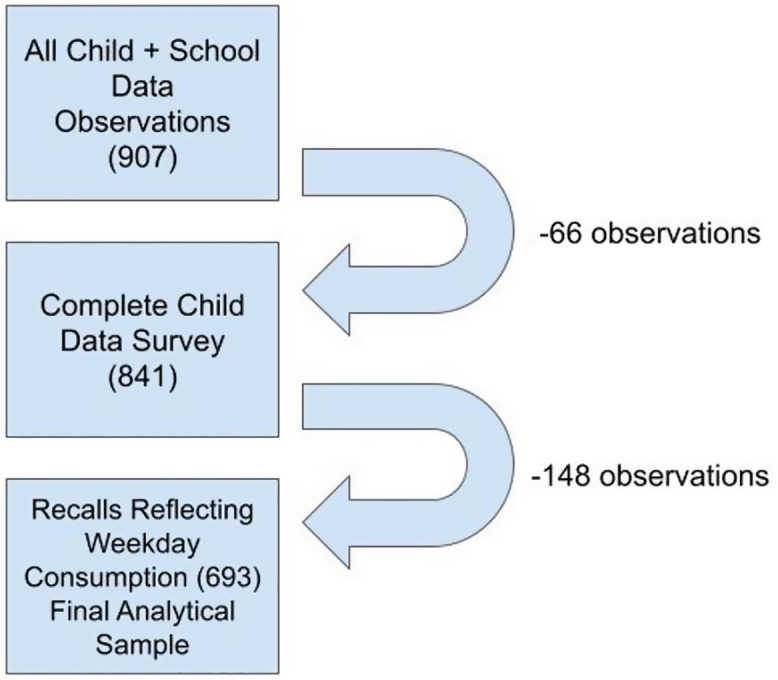
Eligibility Exclusion Criteria.

A total of 1745 primary school children were invited to participate in anticipation of a < 50% response rate. From these responses, 907 children returned surveys, reflecting a 52.0% response rate. The analytical data for this paper was restricted to those with complete data for all included covariates (dropped 66) as well as children’s dietary intake surveys which reflected a school day consumption (dropped 148). This resulted in an analytic sample of data from 693 children (Fig 1), representing an estimated 40.1% of the total student population of the study schools (2214) and 3.66% of children in grades 3–5 across the two parishes (24785). Children’s consumption volumes were capped at 2500 mL, as volumes greater than this could be considered over-reporting by the study population.

### Statistical analysis

All statistical analyses were done using STATA 18.[32] Given that children may not consume all types of beverages in any given day, we used a two-part model. The first part estimates the probability of consumption across beverage categories among all study participants using a probit model. The second part estimates the volume of consumption among consumers using a logistic regression model. The same covariates were used for both parts of the model. We applied a two-part post-estimation using -margins- to find marginal effect estimates on consumption volume across beverage categories of all study participants included in the analysis, consumers and non-consumers. This post-estimation step reverted the log function originally used in the two-part model, and output marginal effect coefficients in mL. This output is shown as the main results. Finally, we performed Wald Chi-Squared Tests to measure the contribution of variable groups to the model fit. A p-value of 0.05 was considered significant. We assumed a null hypothesis that no covariates had significant associations with our three beverage consumption outcomes.

## Results

Descriptive statistics were done on both exposures and outcomes of this study as shown in Table 1. Child demographics and school environment covariates are described by percents and frequencies, while school level indices are described using means and standard deviation.

**Table 1:** Descriptive statistics of Analytical Sample (N=693).

Covariate	Frequency % (N) or Average (SD)
*CHILD DEMOGRAPHICS*	
Child Gender [% (N)]	
*Male*	50.4 (349)
*Female*	49.6 (344)
Child Age (years [SD])	
*7*	0.7 (5)
*8*	23.7 (164)
*9*	32.2 (223)
*10*	34.2 (237)
*11*	9.2 (64)
Child Beverage Knowledge [% (N)]	
*Chose Bagged Drink as Healthier Beverage*	0.7 (5)
*Chose Water as Healthier Beverage*	99.3 (688)
Parental Income [% (N)]	
*Family Income Below Poverty Line (<$7000 JA)*	48.2 (334)
*Family Income Above Poverty Line (>$7000 JA)*	51.8 (359)
Highest Level of Parental Education [% (N)]	
*No Formal School OR Primary School*	12.8 (89)
*All-Age School*	5.5 (38)
*Secondary, High School*	44.6 (309
*Technical/Vocational School*	15.3 (106)
*Tertiary, College/University*	21.8 (151)
*INTERNAL SCHOOL ENVIRONMENT MEASURES*	
School Category [% (N)]	
*Public*	90.6 (628)
*Private*	9.4 (65)
Presence of Indoor Drinking Apparatuses [% (N)]	15.3 (106)
Presence of Outdoor Drinking Apparatuses [% (N)]	77.5 (537)
Industry Marketing Elements and Sponsorships/Donations [Mean (SD)]	10.8 (5.17)
Sweetened Beverage Brands Present Across All Sources [Mean (SD)]	12.7 (6.2)
Water Brands Present Across Sources [Mean (SD)]	3.5 (1.9)
School Meal Related Equipment, Spaces, and School-Sponsored Vending [Mean (SD)]	8.7 (1.7)
Physical Activity Equipment and Spaces [Mean (SD)]	3.3 (1.2)
Outdoor Green Spaces and Non-Physical Activity Related Outdoor Spaces [Mean (SD)]	2.5 (0.7)
Number of Child and Pedestrian Safety Elements [Mean (SD)]	4.7 (1.7)
Number of Sanitation, Trash, and Recycling Apparatuses [Mean (SD)]	3.9 (0.6)
Students Able to Drink Water in the Classroom? [% (N)]	97.0 (672)
*EXTERNAL SCHOOL ENVIRONMENT MEASURES (%)*	
Children Attending Schools Near…	
Urban Residences [% (N)]	42.0 (291)
Paved Walkways/sidewalks [% (N)]	20.5 (143)
Street Vendors [% (N)]	73.4 (509)
Food Stalls [% (N)]	33.0 (229)
Cook Shops [% (N)]	34.2 (237)
Supermarkets [% (N)]	25.5 (163)
Restaurants/Fast Food/Sit Down Establishments [% (N)]	8.7 (60)

The sample reflects near equal distribution of children’s sex, and children with parents above and below the 2018 national poverty line. Slightly less than half of children in the study (44.6%) have parents whose highest level of education is secondary school, and at least 10% of the study population has parents whose highest-level education are one of the following: No Formal School/Primary School (12.8%), Technical/Vocational School (15.3%), and Tertiary, College/University (21.8%).

In the internal school environment, schools had an average of 10.8 food marketing elements which promoted 12.7 sweetened beverage brands and 3.5 plain water brands. Children more frequently attended schools with outdoor drinking apparatuses (77.5%) than they attended schools with indoor drinking water options (15.3%). Regarding school meals, children, on average, attended schools with more than 8 school-meal related equipment, spaces, and indoor school-sponsored vending. External to school campuses, school vendors were the food source which were cited most frequently in the audit (73.4%) compared to food stalls (33.0%), cook shops (34.2%), supermarkets (25.5%), and restaurants/fast food (8.7%).

**Fig 2:**
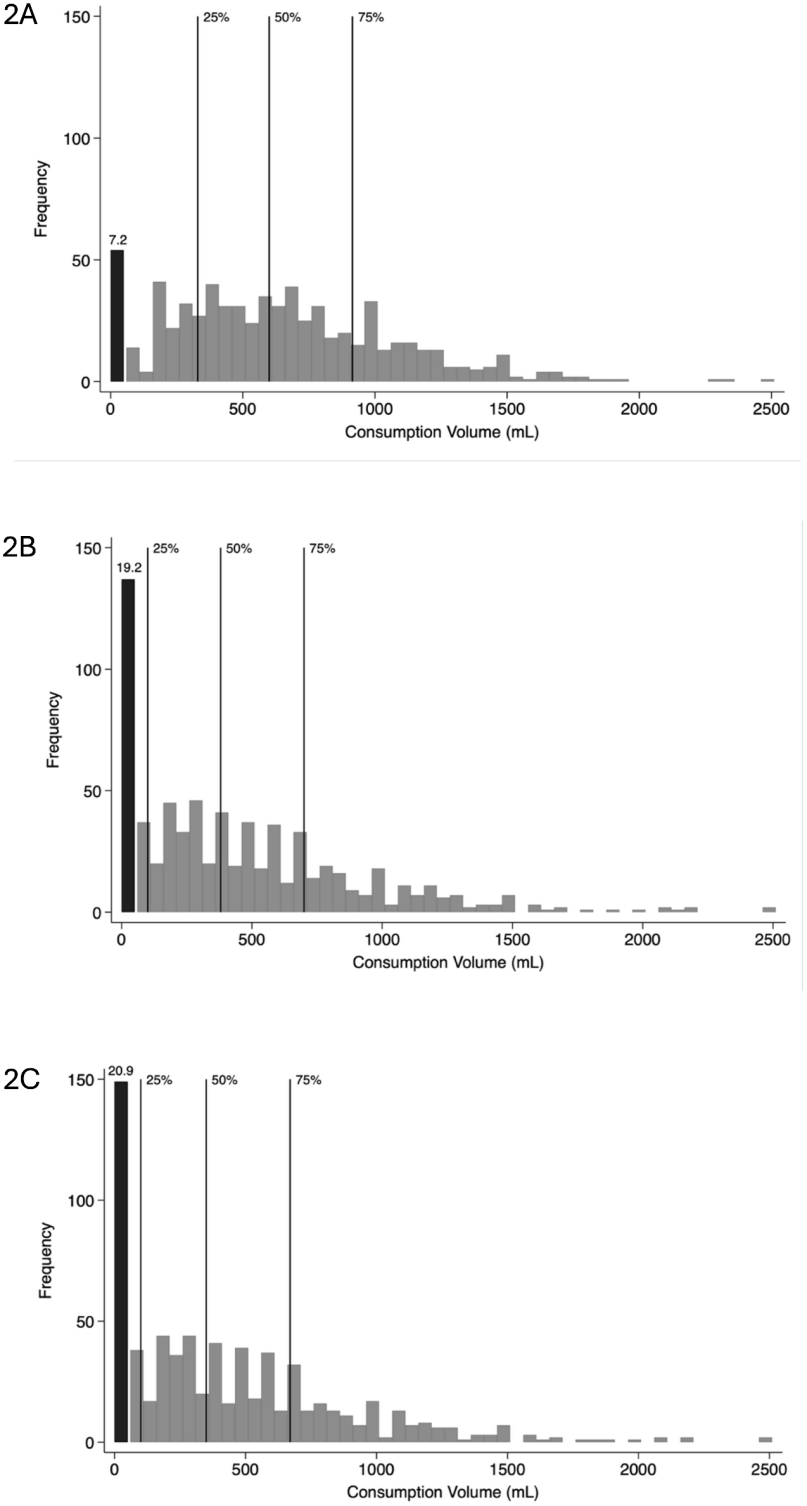
Distributions of children’s consumption of sweetened beverages (2A), unsweetened beverages (2B), and plain water (2C). N = 693. Non-consumers and quartiles are depicted, and the percentage of non-consumers are included above the black bar in each plot.

The beverage intake outcomes of the study are depicted in Fig 2, with the proportion of non-consumers shown in black, as well as quartiles among consumers of each beverage type noted. Across all three beverage types, there is a positive skew, indicating a trend of relatively low amounts of consumption with a small number of children consuming large amounts.

Among consumers of sweetened beverages, 25% consumed less than 400 mL and 75% consumed less than 1000 mL. Relative to a standard can of sweetened beverage (330–355 mL), this is more than one and three cans of drinks, respectively. A relatively low number of children (50, 7.2%) reported consuming either no sweetened beverage.

With regard to the distribution of unsweetened beverages and water consumption, there is a much greater proportion of non-consumers, 19.2% and 20.9, respectively. Given the inclusion of water as an unsweetened beverage and the low consumption of other non-water unsweetened beverages, these two distributions are similar. Among consumers of both types of beverages, 50% consumed less than 500 mL.

As seen in Table 2, as parental education level increases through vocational school training, children’s consumption of sweetened beverages increased. Though children having parents with a college/university education level (the highest measured level) had a lower consumption of sweetened beverages compared to children of parents without formal schooling. With respect to school environment measures, the presence of indoor drinking water apparatuses and the presence of supermarkets near schools both are associated with a significant increase in intake (p < 0.05). The presence of urban residences, while insignificant, was also associated with an increase in sweetened beverage intake.

**Table 2:** Margins Post-Test Estimation and Wald Test Results.

	Sweetened Beverage Intake (mL)		Unsweetened Beverage Intake (mL)		Water Intake (mL)	
Covariates	Marginal effect	SE	Marginal effect	SE	Marginal effect	SE
*CHILD DEMOGRAPHICS*						
Female Child *(ref = male)*	−47.0	35.7	73.3**	37.0	81.9**	36.7
Child Age (year)	15.1	19.2	32.2	19.7	32.1	19.5
Can Identify Water as Healthier than Bagged Drink	−174.9	220.6	−56.1	219.0	−46.1	216.2
Parental Income Above Poverty Line	34.1	39.4	50.6	40.7	52.2	40.4
Parental Education						
*No Formal Schooling/ Primary School*	Ref	Ref	Ref	Ref	Ref	Ref
*All-Age School*	35.5	84.1	110.8	99.5	122.1	96.4
*High School*	93.7*	55.9	−53.4	56.2	−44.5	53.6
*Vocational School*	123.3*	70.6	−83.2	66.5	−61.5	64.9
*College/University*	−74.4	65.6	116.5	77.2	134.3*	76.4
*INTERNAL SCHOOL ENVIRONMENT MEASURES*						
Private School *(ref = public)*	−168.0	106.2	−53.4	108.9	−46.5	107.8
Presence of Indoor Drinking Water Apparatuses	204.9**	76.4	16.7	77.7	13.5	76.8
Presence of Outdoor Drinking Water Apparatuses	−0.8	79.1	−45.8	81.0	−29.8	79.7
# of Industry Marketing Elements and Sponsorships/Donations	−3.0	5.4	−2.1	5.5	−2.8	5.4
# of Sweetened Beverage Brands	5.6	6.3	−2.6	6.5	−2.3	6.4
# of Water Brands	−21.3	31.9	31.1	32.6	37.5	32.5
# of School Meal Related Equipment, Spaces, and School-Sponsored Vending	−9.1	14.0	−3.1	14.3	−8.4	14.2
# of Physical Activity Equipment and Spaces	−29.1	24.6	−12.1	25.3	−8.2	25.0
# of Outdoor Green Spaces and Non-Physical Activity Related Outdoor Spaces	−8.2	42.8	2.7	43.9	3.8	43.3
# of Child and Pedestrian Safety Elements	21.0	20.1	−7.1	20.5	−13.3	20.3
# of Sanitation, Trash, and Recycling Apparatuses	10.3	56.4	43.7	58.2	34.5	57.6
Students Able to Drink Water in Class?	−121.4	119.4	71.8	156.4	59.6	153.4
	**Sweetened Beverage Intake (mL)**		**Unsweetened Beverage Intake (mL)**		**Water Intake (mL)**	
**Covariates**	**Marginal Effect**	**SE**	**Marginal effect**	**SE**	**Marginal effect**	**SE**
*EXTERNAL SCHOOL ENVIRONMENT MEASURES*						
Presence of Urban Residences Near Schools	124.3*	66.4	86.2	66.5	96.7	65.9
Presence of Paved Roads Near Schools	88.4	76.2	94.1	78.8	79.9	77.8
Presence of Food Vendors Near Schools	−17.0	95.5	−25.9	97.6	1.4	96.4
Presence of Food Stalls Near Schools	31.7	81.6	−111.5	83.3	−124.4	82.5
Presence of Cook Shops Near Schools	91.1	65.3	18.0	66.7	14.4	65.7
Presence of Supermarkets Near Schools	202.0**	65.4	2.3	66.6	16.1	65.9
Presence of Restaurants/Fast Food/Sit Down Establishments Near Schools	−109.6	100.7	−92.7	101.5	−110.2	100.6

**Wald Test of joint significance**	**Chi** ^**2**^	**P > Chi** ^**2**^	**Chi** ^**2**^	**P > Chi** ^**2**^	**Chi** ^**2**^	**P > Chi** ^**2**^
Child demographics	23.82	0.05*	26.31	0.02**	28.66	0.02**
Internal School Environment Measures	31.97	0.08*	20.62	0.66	20.03	0.70
External School Environment Measures	35.14	0.00**	16.31	0.30	14.98	0.38
						

** p < 0.05, * p < 0.1

Our model showed no single environmental covariate being significantly associated with unsweetened beverage intake. Among child-level demographics, we found that female students in our sample consumed more unsweetened beverages than their male counterparts.

The water intake model closely resembled the unsweetened beverage model, indicating a low consumption of non-water unsweetened beverages. Similarly, being female was significantly associated with an increase in intake. Having at least one parent or guardian of the highest education level was shown to have a weak correlation with consumption of more water (p < 0.1).

Wald Tests showed that, child demographic factors were jointly significant in being associated with all three beverage types, while grouped external school environment measures were proved only significant with students’ sweetened drink intake.

The table in S1 Table provide the results for each part of the two-part models, revealing some potential factors that may drive the probability of consuming select beverages. We found that a classroom’s policy of preventing drinking water in the classroom perfectly predicted a student’s probability of consuming sweetened beverage.

## Discussion

This cross-sectional study sought to measure the relationships between child and school level influencers on Jamaican children’s beverage intake, providing evidence that child demographic and external school environment measures have significant associated with volumes of beverages consumed.

Evaluating sweetened and unsweetened beverage intake, the results showed varying associations of modeling covariates on consumption. At the child level, gender seemed to play a role, with female school children drinking more water and other unsweetened beverages than male school children. Although, the two groups drank indistinguishable amounts of sweetened beverages, aligning with other regional and global trends.[15] Parental education appeared to be loosely correlated to consumption with children of parents at the highest level of education drinking more water than children of parents with no formal schooling, though this correlation was not significant at conventional levels. Across all three beverage types, child-level measures were shown to jointly contribute to beverage intake model fit.

Internal and external school metrics showed varied associations on beverage intake. The presence of indoor drinking water and supermarkets near schools proved to be the only significant covariates associated with sweetened beverage intake, both correlated with increases. Wald Tests provided sufficient evidence to reject the possibility that external school environment covariates are jointly insignificant in contributing to the sweetened beverage outcome, meaning that their interaction may have a role in children’s choice to drink these sweetened drinks.

Distinguishing between consumers and non-consumers helps to interpret the differences between sweetened and unsweetened beverage consumption patterns. More than a fifth of the children included in the sample did not consume any water during the school day, a stark comparison to the 7.2% of sweetened beverages non-consumers. This difference in overall drinking habits may be related to students’ access to water during the school day. The initial probit model defines, unsurprisingly, that students who were permitting drinking water in the classroom did drink water more often during school days. Yet, even more striking, was that every student who was in a classroom in which drinking water was prohibited consumed sweetened beverages. Generally, children are encouraged to bring water containers into the classroom, though the results of school surveys show that they may be unable to refill these containers throughout the day.

Interactions between classroom rules, school wide policies, and the internal school environment could also contribute to overall patterns in beverage choice. Nearly all students in the study sample were able to consume water in the classroom, yet only 15.3% of them attended schools with indoor water access points (see Table 1). Previous work has shown associations between district-wide policies on beverage consumption, particularly that of sweetened drinks. Policies restricting both the presence of these drinks and the advertisement of them within schools has been shown to be associated with lower odds of regular sweetened drink consumptions.[33] Furthermore, our study found similar results to others in that an increase in the presence of healthier beverage alternatives was not associated with a decrease in sweetened beverages consumption.[33,34]

Previous work among global populations have shown that a range of interventions can impact children’s beverage consumption in school.[35–38] Interventions, including both environmental and educational types, have been shown to decrease SSB intake, with combined approaches being particularly effective.[38] While children in this study seem to have a high level of beverage knowledge, an intervention that engages parents and teacher may help strengthen the influence of beverage knowledge on actual beverage choice. Previous work has shown that engaging parents can significantly decrease the consumption of sodas and other SSBs.[39,40]

Beyond the physical environment, social environments at schools have also been shown to influence young children’s dietary patterns. A peer mediated behavioral study in the Caribbean showed that peer influencers can help reduce other school children’s consumption of SSB, while also potentially increasing their water intake.[41] Children and adolescents’ behavior can be influenced more by their friends or peers, especially in school settings, than their parents, and thus is important to account for in any future school-based interventions in this setting.[42]

A recently approved national school nutrition policy in Jamaica seeks to regulate nutritional standards within school and manage the internal and external food environments. Though the presence of external food sources was low in this survey, the quality of their provisions is largely unregulated. With a large focus oversight of school food vendors, this policy may help improve the quality of beverages students consume. The country’s school beverage guidelines, which increasingly limits the amount of allowed sugar since 2018, are not necessarily supported by adequate policy structures and has allowed for the provision of unhealthy beverages both on and near school campuses. A regional public health collaborative has provided a framework for these limitations, encouraging not only the prohibition on unhealthy drinks, but also the availability and promotion of healthier options.[43]

There is precedence for formal policies that limit the sales of SSBs in other Caribbean countries, namely in Bermuda, Barbados, and Trinidad and Tobago. In 2006, Bermuda banned the sale of SSBs in schools and in 2018, implemented a tax on sugary drinks and food in 2018.[44,45] This resulted in a nearly half of the population consuming fewer taxes goods, including drinks. Barbados implemented a similar SSB tax in 2015 which led to both a decrease in SSB sales, as well as an increase in unsweetened beverage purchases.[22] In 2017, St. Lucia’s Ministry of Health and Ministry of Education moved forward with a ban on the sale of SSBs in schools, though there is limited evidence on the effectiveness of this ban.[44]

Given the low presence of policies regulating nutrition and dietary components in Jamaican schools and the challenges of enforcing these types of policy interventions, a synergistic legislative framework is needed to drive effective change.[4,46,47] A suite of such policies must be multisectoral, improving the quality of food and beverage marketing in schools, offering healthy nutrition education opportunities, and modeling desired consumption behavior.[46,48,49] Programs improving children’s diet in school will have the greatest benefit if closely tied to other related policies and legislation, such as those touching upon social protections, health, agriculture, and urban food planning.[50] While individual policies may falter or be difficult to enforce, a coherent set of legislative implementation can lead to better student health outcomes. [51,52]

### Strengths and limitations

Strengths of this study include extensive school auditing measures of urban schools in the greater Kingston metro area. Information on both internal and external school food environments is rare, and to our knowledge, there is not another dataset describing similar information in Jamaica. The breakdown of consumer and non-consumer patterns of multiple beverage types also allowed for a better understanding of children’s consumption at school and factors that may influence the basic decision to drink or not drink a certain beverage type. Children in the study had relatively well mixed social demographics, and continuity of the research team contributed heavily to study-specific troubleshooting questions as well as context-specific analyses.

Limitations of this study included the secondary analysis nature of the investigation. There were several cases where survey/audit questions could have been more elaborate, such as familial socio-economic status, beverage knowledge, and internal school food provisions. A single adjusted 24-hour recall survey focused on beverages and select foods may not fully describe children’s usual drinking habits due to daily variation but was most practical to collect among this age of school children. While this analysis included primary schools in the Kingston metro area, many other parts of the country are not reflected and may show different relationships between consumption habits and school food environments. Lastly, the data from 2018 represents beverage intake and SFE prior to the COVID-19 pandemic. While school environment data was collected in 2022, children’s dietary intake was not, and thus the two were not linked nor included in this evaluation.

## Conclusion

Through a cross-sectional analysis of Jamaican school children’s sweetened and unsweetened beverage consumption, we described the differences and potential child-level and school-level influencers of in-school drinking habits. Gender was shown to be significantly associated was differences in unsweetened beverage intake, while the presence of indoor drinking water apparatuses and supermarkets near schools were associated with variations in sweetened beverage consumption. Collectively, external school environment factors and child-level demographics improved the fit of sweetened and unsweetened models, respectively. The ability of students to drink water in the classroom and schools likely influenced sweetened beverage intake. Schools can improve their student overall beverage intakes by implementing additional policies to decrease the presence and marketing of SSBs in and around schools. These types of policies and other interventions to improve children’s beverage intake should be considered and incorporated in the implementation of Jamaica’s nation school nutrition policy and other intervention to better school children’s nutritional health.

## Supporting information

S1 Table Results of Sweetened Beverage, Unsweetened Beverage, and Water Intake Two-Part Models. (ZIP)
